# Cryptic genetic diversity and associated ecological differences of *Anastatus orientalis*, an egg parasitoid of the spotted lanternfly

**DOI:** 10.3389/finsc.2023.1154651

**Published:** 2023-06-02

**Authors:** Yunke Wu, Hannah J. Broadley, Kendra A. Vieira, John J. McCormack, Corrine A. Losch, Hyeban Namgung, Yeongmo Kim, Hyojoong Kim, Alana R. McGraw, Marjorie Z. Palmeri, Seunghwan Lee, Liangming Cao, Xiaoyi Wang, Juli R. Gould

**Affiliations:** ^1^ Forest Pest Methods Laboratory, United States Department of Agriculture, Animal and Plant Health Inspection Service, Plant Protection and Quarantine, Science and Technology, Buzzards Bay, MA, United States; ^2^ Department of Ecology and Evolutionary Biology, Cornell University, Ithaca, NY, United States; ^3^ Department of Environmental Conservation, University of Massachusetts, Amherst, MA, United States; ^4^ Department of Biological Science, Kunsan National University, Gunsan, Jeonbuk, Republic of Korea; ^5^ Department of Entomology, Kansas State University, Manhattan, KS, United States; ^6^ Insect Biosystematics Laboratory, Department of Agricultural Biotechnology, Seoul National University, Seoul, Republic of Korea; ^7^ Research Institute for Agricultural and Life Sciences, Seoul National University, Seoul, Republic of Korea; ^8^ Key Laboratory of Forest Protection of National Forestry and Grassland Administration, Ecology and Nature Conservation Institute, Chinese Academy of Forestry, Beijing, China

**Keywords:** spotted lanternfly, biological control, Eupelmidae, genetic diversity, life history, iso-female lines

## Abstract

*Anastatus orientalis*, native to northern China, is an egg parasitoid wasp of the spotted lanternfly (*Lycorma delicatula*) and is being tested as a potential biological control agent for invasive *L. delicatula* in the United States. As a component of these evaluations, live *A. orientalis* collected from Beijing and Yantai in China were reared in containment in the U.S. These specimens showed different responses in diapause behaviors to rearing conditions used previously by other researchers. To understand the primary mechanism potentially driving discrepancies in important life history traits, we used molecular tools to examine the genetic composition of *A. orientalis* from China and from South Korea, where the parasitoid has been introduced to aid in the population management of invasive *L. delicatula*. Molecular analysis of mitochondrial DNA recovered six haplotype groups, which exhibit biased frequency of abundance between collection sites. Some haplotypes are widespread, and others only occur in certain locations. No apparent pattern is observed between wasps collected from different years or emergence seasons. Uncorrected genetic distances between haplotype groups range from 0.44% to 1.44% after controlling for within-group variation. Genetic variance of *A. orientalis* is characterized by high levels of local diversity that contrasts with a lack of a broad-scale population structure. The introduced Korean population exhibits lower genetic diversity compared to native populations. Additionally, we created iso-female lines for major haplotype groups through laboratory rearing. Differences in diapause behavior were correlated with mitochondrial haplotype. Our results indicate that the observed life history traits in *A. orientalis* have a genetic base.

## Introduction

The spotted lanternfly, *Lycorma delicatula* White (Hemiptera: Fulgoridae), is a destructive invasive insect in North America. It is a highly polyphagous planthopper feeding on over 170 species of plants across 33 families (1). Its preferred host plant is the tree of heaven (*Ailanthus altissima*), which is also invasive in the United States, but *L. delicatula* is considered a high-risk pest of grapes and hops with the potential to impact fruit trees, walnuts, ornamentals, hardwood, forest, and shade tree species (2). *Lycorma delicatula* is native to China (3), where populations are typically relatively low in density, and it is not a significant pest (4). To control the invasive population of *L. delicatula* in the Unites States, efforts have been made to identify its natural enemies in the native range. An egg parasitoid wasp, *Anastatus orientalis* Yang & Choi (Hymenoptera: Eupelmidae), has been identified as a promising candidate for the biological control of *L. delicatula* (4). Previous work has shown that *A. orientalis* demonstrates strong attraction to *L. delicatula* (5) and has a high attack rate on *L. delicatula* in its native range (typically between 30%–40%, 4, 6; but as high as 80% in 7). *Anastatus orientalis* was introduced to South Korea where it is being used as a management strategy for the control of invasive *L. delicatula* (8), where population density seems to be suppressed as a result of the introduction. Extensive studies are currently being conducted with *A. orientalis* to consider it as a potential biological control agent for invasive *L. delicatula* in the U.S.

It has been reported that Chinese and South Korean researchers were able to continuously rear *A. orientalis* for at least eight generations under 25°C and long-day conditions (6, 9). However, a recent study that evaluated progeny production under the same reported conditions found that, in contrast, nearly all *A. orientalis* larvae went into apparent diapause (10). The parent generations of those larvae were collected in Beijing, China, as were those in the earlier studies. However, when reared under temperature and daylight conditions that mimicked Beijing in the fall, nearly all larvae emerged from the host eggs without diapause (10). The observed discrepancy leads to speculation that differences exists between the strains or lineages of *A. orientalis*.

It has been documented that genetic factors play an important role in the plasticity of life cycles in insects, particularly diapause, which is arguably the most important adaptative strategy to face seasonal environmental heterogeneity (11–13). For example, differential gene expression could regulate insect diapause at the transcriptional level (14, 15). Variable life cycle traits in insects can also be directly inherited by progeny from parents, which may lead to genetically differentiated strains maintained by selection (16). Some traits are mainly under the control of multiple genes through epistasis, such as the differential photoperiodic response of the pitcher-plant mosquito (*Wyemyia smithii*) (17). Other traits can be affected by a single segregating locus on the sex chromosome, like the voltinism and pheromone pattern in the European corn borer moth (*Ostrinia nubilalis*) (18). Furthermore, different genetic strains can exhibit physiological differences such as the level of virus-resistance in biotypes of coconut rhinoceros beetle (*Oryctes rhinoceros*), which has significant implications for pest management (19).

An initial survey of the standard mitochondrial COI barcode of *A. orientalis* in northern China suggested variable diapause behaviors among different geographic populations (20). However, using 48 specimens sampled from five locations, this study did not find a distinct population structure but did find that within-location variations dominated the overall genetic variance (20). In the current study, we aimed to further understand cryptic genetic differentiation within this parasitoid, which appears morphologically conserved across its range, and assess the association between genetic lineages and life cycle characteristics. We analyzed a larger number of *A. orientalis* specimens collected in multiple years along with additional samples from South Korea. We also designed new species-specific COI barcode primers and added a second mitochondrial DNA fragment downstream from the standard barcode sequence. The expanded sampling and sequence data in the current study provides new insights into the genetic diversity of *A. orientalis* both in its native and introduced range. In addition, we created iso-female lines based on the genetic results and showed the impact of genetics on diapause behaviors. We also evaluated rearing conditions to maximize insect production in the event that *A. orientalis* is selected as the biological control agent for spotted lanternfly.

## Materials and methods

### Insect material collection

Parasitized *Lycorma delicatula* egg masses were collected from China and South Korea between 2019–2021. In China, *L. delicatula* egg masses were collected during the winter and spring from two locations: Beijing and Yantai (Shandong province), from areas where, based on prior knowledge, some parasitism was expected by *Anastatus orientalis.* Three collections were made in Beijing between 2019–2021 and two in Yantai from 2020–2021. In addition, we collected *L. delicatula* egg masses around three cities in South Korea (Nonsan, Anseong, and Buyeo) between March and April 2021. *Anastatus orientalis* that emerged from those egg masses were included in the molecular analysis. Egg masses were carved from the bark of tree trunks with a small knife, stored in locked food boxes by locations at room temperature, and shipped using appropriate permits to the Forest Pest Methods Laboratory (FPML) Insect Containment Facility, U.S. Department of Agriculture in Massachusetts. Voucher specimens were stored in 95% ethanol for molecular analysis (**Table 1**).

**Table 1:** Sampling locations in China and South Korea.

Country	City	Collection time	n	h	S	Hd	π
China	Beijing (N 39.9925°, E 116.2109°)	2019	33	6	14	0.703	0.0065
		2020	29	7	17	0.685	0.0061
		2021	30	4	12	0.561	0.0061
	Yantai (N 37.3570°, E 121.4028°)	2020 Spring	7	4	19	0.714	0.0079
		2020 Fall	15	4	17	0.667	0.0075
		2021 Spring	30	5	21	0.692	0.0079
		2021 Fall	16	4	18	0.442	0.0052
South Korea	Buyeo (N 36.3184°, E 126.8451°)	2021	80	4	11	0.601	0.0060
	Anseong (N 36.9373°, E 127.2670°)	2021	42	3	12	0.180	0.0022
	Nonsan (N 36.1662°, E 127.1914°)	2021	12	2	11	0.303	0.0037

Population genetic statistics include the number of sequences (n), number of unique haplotypes (h), number of polymorphic sites (S), haplotype diversity (Hd), and nucleotide diversity (π).

### Genomic DNA extraction

We applied two DNA extraction methods to minimize damages to the morphology of each adult wasp, which can be used later for morphological comparison. The process started with a crude DNA extraction, where individual insect was submerged in 100 µl of the prepared extraction buffer that included proteinase K (the ProtK 21) and incubated at 37°C overnight. The reaction was deactivated the next morning by heating the buffer to 75°C for 30 minutes on a heat block. The intact wasp was removed from the buffer to be stored separately. The DNA extract was then cooled and stored at -20°C for subsequent use. When the crude DNA extract failed to yield any PCR product, we revisited the specimen and pulled a single leg, which was processed with the QIAGEN DNeasy Blood & Tissue Kit (QIAGEN, Germantown, MD) following the manufacturer’s protocol, except that purified DNA was eluted in 100 µl buffer. South Korean wasps were all processed with the QIAGEN kit.

### Primer design, PCR, and sequencing

We first attempted to amplify the mitochondrial COI barcode for *A. orientalis* using the universal primers LCO1490/HCO2198 (22) as has previously been done (20). However, some specimens yielded no PCR product, and even among those with a successful PCR, the sequencing reaction resulted in mostly noisy data. Upon close examination, an 11-T (thymine) repetitive region near the 5′ end of the barcode was identified as the cause of frame shifts during the extension phase of DNA the sequencing that resulted in noisy data downstream. Therefore, we designed a new primer pair (forward 192F: 5′-TTGGGAATTATTTTGTTCCA-3′; reverse 720R: 5′-TGAGAAATCAATCCAAATCC-3′) to circumvent the repetitive region and maximize amplification/sequencing success for *A. orientalis*. The primer pair amplified a 529 bp fragment that overlapped with 70.1% of the full COI barcode. To further increase the amount of data, we amplified an additional 437 bp fragment immediately downstream from the COI barcode using a second pair of universal primers known as NJ/MD with minor modifications (forward NJ: 5′- TATATTTTAATTTTRCCTGGATTTGG-3′, modified 23; reverse MD: 5′- ATTGCAAATACTGCACCTAT-3′; 24), which demonstrated its usefulness in parasitoid wasps (25).

PCR amplification was conducted in a reaction mix (20 µl total volume) containing 9 µl of molecular grade water, 2 µl of 10X PCR buffer without MgCl_2_, 2.8 µl of MgCl_2_ (25 mM), 3.2 µl of dNTP solution (1.25 mM), 0.4 µl of forward and reverse primers (10 pmol/µl), 0.2 µl of JumpStart taq DNA Polymerase (2.5 units/µl), and 2 µl of DNA template. The primer pair 192F/720R was amplified under the following cycling condition: initial denaturation at 94°C for 3 min, followed by 35 cycles of denaturation at 94°C for 30 s, annealing at 54°C for 30 s, extension at 72°C for 1 min, and a final extension at 72°C for 10 min. Cycling condition for the primer pair NJ/MD consisted of initial denaturation at 93°C for 3 min, followed by 35 cycles of denaturation at 93°C for 15 s, annealing at 46°C for 45 s, extension at 68°C for 45 s, and a final extension at 68°C for 7 min. All PCRs included negative control for monitoring contamination. Amplified PCR products were examined on 3% agarose gels, purified by ExoSAP-IT (Affymetrix, Cleveland, OH) following the manufacturer’s protocol, and then sequenced on an ABI 3730XL (ACGT, Inc., Wheeling, IL).

### Phylogenetic analysis

We used Geneious Prime 2021.1.1 (https://www.geneious.com) to edit chromatograms and perform sequence alignment with the MUSCLE algorithm under default parameters. Genetic diversity statistics were calculated for Beijing, Yantai, and South Korean specimens using DNAsp v6 (26), including the number of unique haplotypes (h), number of polymorphic sites (S), haplotype diversity (Hd), and nucleotide diversity (π) (**Table 1**). Uncorrected *p*-distances were calculated between haplotype groups, sampling years, and sampling locations in MEGA 11 (27). Mitochondrial genealogies were reconstructed using maximum likelihood (ML) analysis and Bayesian inference. *Anastatus fulloi* was chosen as the outgroup because it is the only species with both 192/720 and NJ/MD data available and also native to China (28). The best-fit nucleotide substitution model was selected by the corrected Akaike Information Criterion (AICc) in jModeltest 2.1.7 (29). The ML analysis was carried out by RAxML v8.2.11 (30) implemented in Geneious, using the best-fit substitution model with the algorithm that simultaneously searches for the best-scoring ML tree and does rapid bootstrapping. A parsimony tree was used as the starting seed for ML tree searching. Nodal support values were estimated through 500 non-parametric bootstrap replicates (BP). The Bayesian inference was carried out by MrBayes 3.2.6 (31) also implemented in Geneious using the best-fit substitution model with two independent runs, each of which had four heated chains running for ten million generations. Tree subsampling frequency was set to every 10,000 generations and the first 25% of trees were discarded as burn-in. Posterior probability (PP) was used to evaluate nodal support. To visually display connection between haplotypes, haplotype frequencies, and how haplotypes were shared between sampling years and locations, we further reconstructed a statistical parsimony network (TCS network) using PopART 1.7 (32).

### Establishing iso-female lines

To build pure colonies of the three most common *A. orientalis* haplotypes that we detected, Haplotypes B, C, and D (see Results), we reared out and genotyped (see methods above) a subset of wasps from our live collections of *A. orientalis* from China described above and maintained the lines separately in colony. To start these lines, in April 2020, 100 *L. delicatula* egg masses were collected from the field in Beijing, China and 100 from Yantai, China and shipped to the USDA APHIS Forest Pest Methods Laboratory’s Insect Containment Facility. Egg masses from Beijing were held in a growth chamber (Percival, Perry, Iowa) under conditions that simulated real-time temperature and light conditions in Beijing, China as conditions cycled from winter to spring, summer, fall, and winter conditions again. Hereafter, we refer to these conditions as “Beijing-match conditions”. Similarly, the egg masses from Yantai were held under conditions that simulated temperature and light conditions in Longkou, Yantai, hereafter referred to as “Yantai-match conditions”. Temperature data used were 11-year hourly averages (2007–2017) for each location from the NOAA National Centers for Environmental Information (Global Hourly – Integrated Surface Database). Chamber temperature conditions adjusted each hour to match the hourly averages obtained from the Integrated Surface Database. Light data (daily sunrise, sunset, and civil twilight times) were obtained from timeanddate.com. A relative humidity of 65% was maintained in both chambers. In the fall, on the date when maximum temperatures did not exceed 10°C (November 10 for Beijing and November 22 for Yantai), the chambers were set to constant 5°C with no lights for overwintering. In the spring, on March 7 for both Beijing and Yantai, the chambers resumed tracking the hourly temperature averages and daily light conditions. The full datasets used to program the chambers for “Beijing-match conditions” and “Yantai-match conditions” are available as a supplement (**Supplementary Table 1**). We collected emerging adult wasps three times per week (Monday, Wednesday, and Friday) for subsequent analysis, rearing, and experiments.

To develop a Haplotype B line, F1 progeny of wasps from April 2020 collections of parasitized egg masses from Yantai were collected three days per week (Monday, Wednesday, and Friday) in September 2020 so that the wasps were ≤ 72 hrs old when collected. Groups of up to five males and 15 females were placed in medium-sized rearing containers (473 ml plastic deli cup, SD16 GenPak, Charlotte, NC), which were modified to include a mesh lid. Wasps were provided with a streak of pure honey and held in Yantai-match conditions without access to egg masses for a mating and preoviposition period of five to nine days. Following preoviposition, 60 female *A. orientalis* were placed individually in small rearing containers (118 ml or 177 ml plastic deli cup, AD04 or AD06, GenPak, Charlotte, NC) modified to include a mesh lid. Each female wasp was provisioned with one *L. delicatula* egg mass and honey and remained in Yantai-match conditions for a one-week exposure. The *L. delicatula* egg masses had been collected from January to March of the same year in Lancaster County, Berks County, and Lebanon County, Pennsylvania. Prior to use, the egg masses were held in constant 5°C conditions with no lights to limit development. Parasitism of field collected eggs has been found to be extremely rare (unpublished data), and no or at least inconsequential numbers of *Anastatus* wasps native or resident to the U.S. would have been present in egg masses used for this study.

Following one week of exposure to the wasp, each egg mass was moved to its own cup. Each wasp was then provided with a second egg mass for a second exposure week and a third egg mass for a third exposure week. Following the third exposure week, each female wasp was preserved in 95% ethanol and genetically analyzed to determine haplotype. Egg masses that had been exposed to Haplotype C and D wasps were discarded. Egg masses exposed to Haplotype B wasps (16 out of the 60 total wasps) remained in Yantai-match conditions for approximately one month (33–35 d), after which they were moved to 25°C long-day conditions to promote emergence. No significant emergence occurred after one month in 25°C conditions, and the egg masses were moved back into Yantai-match conditions for overwintering. The egg masses remained at Yantai-match conditions until their emergence in September 2021, at which point they were reared as described below.

To build pure colonies of Haplotypes C and D, 55 F2 *A. orientalis* female progeny of wasps from Beijing were held in Beijing-match conditions in May 2021, allowed one week to mate and develop eggs, and then provisioned with egg masses to produce progeny. The wasps were provided with new egg masses for a second exposure week, after which they were preserved in 95% ethanol and genetically analyzed. Egg masses exposed to Haplotype C (16 wasps) and Haplotype D (39 wasps) were labeled accordingly and held in Beijing-match conditions until their emergence in September 2021, at which point they were reared as described below.

### Evaluation of rearing conditions

Adult *A. orientalis* from all three iso-female lines (Haplotypes B, C, and D) began emerging in September 2021. Adult wasps of each haplotype were collected three days per week (Monday, Wednesday, and Friday) and combined in groups separated by haplotype of up to five males and 15 females in medium-sized rearing containers (containers described above). Wasps of each haplotype (more than 40 of each) were then moved into environmental conditions that mimicked mid-September in Beijing, China (referred to as “Beijing-fall conditions”) and another subset was moved to 25°C long-day conditions. Beijing-fall conditions cycled daily from a high of 25°C and a low of 14°C, lights on 5:55 AM to 6:23 PM, and 65% RH. The 25°C long-day condition maintained a constant 25°C temperature, 65% RH, and 17.5:6.5 (L:D) h (lights on 6:00 AM to 11:30 PM). Note however, that prior work with 25°C conditions set 16:8 (L:D) h with lights on from 6:00 AM to 10:00 PM. In this study, the lights were on 1.5 hrs longer due to a technical issue with the timer. The full datasets used to program the chambers for “Beijing-fall conditions” and “25°C long-day conditions” are available in **Supplementary Table 1**. A diagram depicting the full rearing protocol is also available in **Supplementary Figure 1**. These two temperature conditions were selected because Beijing-fall conditions had previously been identified as an effective rearing condition for *A. orientalis* Haplotype C (10) and 25°C long-day conditions had been identified as an effective rearing condition for *A. orientalis* (unknown haplotype) (6, 9).

The wasps were held in each rearing condition without access to egg masses for a one-week preoviposition period. Following preoviposition, one female *A. orientalis* was placed in a small rearing container (118 ml or 177 ml plastic deli cup, AD04 or AD06, GenPak, Charlotte, NC or 237 ml deli cup, 6011 NYHI, Canada) with one *L. delicatula* egg mass and a streak of honey for a one-week exposure. Egg masses of a comparable size (approximately 40-45 eggs per egg mass) were selected across replicates. Wasps remained in their respective rearing conditions (Beijing-fall or 25°C) for exposure. Following wasp exposure, each egg mass was moved to its own cup, and the wasp was provided with another *L. delicatula* egg mass for a second one-week exposure. Wasps were removed after the second exposure week and saved in 95% ethanol. The resulting parasitized egg masses were held for one month (28 d) in their respective rearing conditions to allow for wasp development. Replication of 80 egg masses for each haplotype in each exposure condition (an extra replicate was run for Haplotype B in Beijing-fall). Egg masses in the Beijing-fall condition were then moved to 25°C conditions to promote emergence as described by Broadley et al. (10). Egg masses assigned to 25°C conditions remained at 25°C conditions for emergence. The number of male wasps, female wasps, and *L. delicatula* nymphs that emerged were recorded three times per week (Monday, Wednesday, and Friday). All egg masses were allowed eight weeks (56 d) in 25°C conditions for emergence before they were discarded.

### Statistical analysis

A two-way ANOVA with haplotype and rearing condition as factors and the interaction between these two factors on number of progeny produced as the response and another two-way ANOVA with the duration of emergence as the response were run. Replicates in which the parent wasp died during egg mass exposure were not included in the analysis; these accounted for a small number of replicates with only 1 to 9 replicates per each combination of haplotype and rearing condition. To test for an effect of haplotype or rearing condition on the resulting proportion of female progeny, we used a generalized linear model (GLM) with a binomial distribution and logit link. All statistical analyses were run using JMP 13.1.0 (SAS Institute Inc.).

## Results

### Molecular phylogenetic analysis

The ProtK DNA extraction method worked well for adult *A. orientalis* specimens, generating positive PCR amplifications for more than 80% of the samples that remained intact. Samples that failed the ProtK method had one leg removed, which was processed by the QIAGEN kit. In total, sequence data were generated for 160 A*. orientalis* collected in China (92 from Beijing and 68 from Yantai) and 134 collected in South Korea for the 192/720 and NJ/MD fragment. The two fragments overlapped by 38 bp and therefore were combined into a single 928 bp sequence within the span of the mitochondrial COI gene. No insertion/deletion nor premature stop codons were observed, as expected given its protein-coding function. After trimming both ends, the final sequence alignment included 900 bp. Population genetic statistics were provided in **Table 1**.

**Figure 1:**
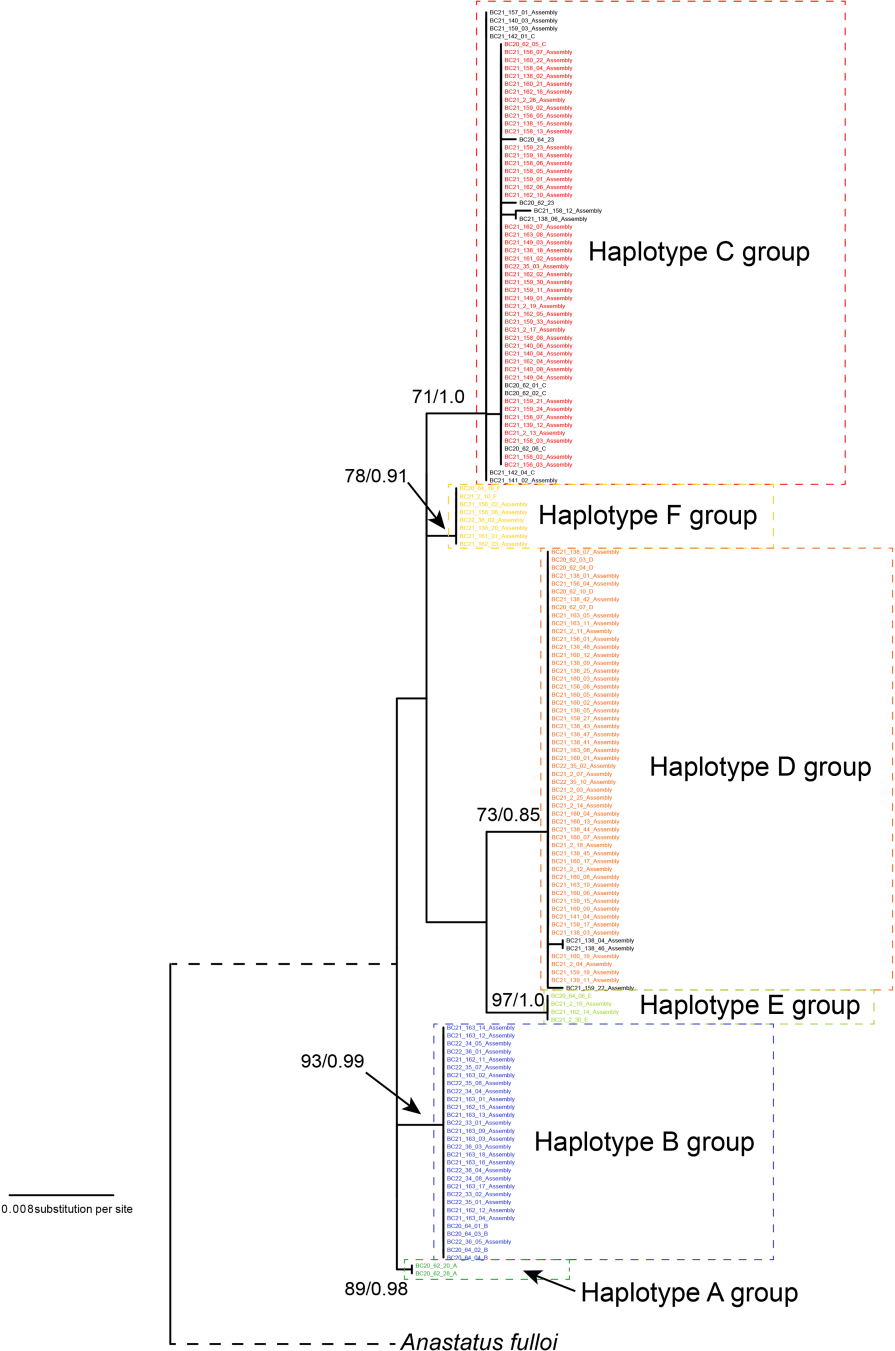
Maximum likelihood (ML) gene tree of *A. orientalis* collected from China. Tree is rooted by *A. ulloi*. The six haplotype **(A–F)** are color-coded and haplotype groups are designated by dashed lines. Samples in black color represent haplotypes that differ by a few substitutions from their respective main haplotypes. Numbers on branches are nodal support: ML bootstrap values/Bayesian posterior probabilities.

We reconstructed the gene tree based only on the Chinese specimens, as the South Korean population was secondarily introduced from China. The best-fit nucleotide substitution model was selected as the GTR+G model. The ML and Bayesian analysis produced identical haplotype groupings, albeit relationships between groups remained unresolved since branch supports were low; only the ML tree is presented here (**Figure 1**). We identified six well-differentiated haplotype group (A–F), three of which (i.e., groups B, C, D) appeared in high frequencies and the other three (A, E, and F) were much rarer. All unique haplotypes were deposited in GenBank (accession numbers, OQ555811-OQ556104). The inferred phylogenetic relationships among those haplotype groups remained unclear as nodal support values were relatively low (ML BP < 80, Bayesian PP < 0.95). The maximum uncorrected *p*-distance between individual specimens in the Chinese *A. orientalis* was 1.56%, which can be found between groups C and E. After controlling for small within-group variations, net mean *p*-distance between haplotype groups ranged from 0.44% to 1.44% (**Table 2**).

**Table 2:** Uncorrected p-distances between haplotype groups (lower diagonal) and within each group (diagonal).

	Group A	Group B	Group C	Group D	Group E	Group F
Group A	0.0000					
Group B	0.0044	0.0000				
Group C	0.0085	0.0108	0.0008			
Group D	0.0122	0.0144	0.0115	0.0001		
Group E	0.0122	0.0133	0.0139	0.0089	0.0000	
Group F	0.0056	0.0078	0.0055	0.0111	0.0111	0.0000

Group C, D and F are shared between Beijing and Yantai, while group A is restricted to Beijing and group B and E are endemic to Yantai. Group C and D are found in South Korea.

**Figure 2:**
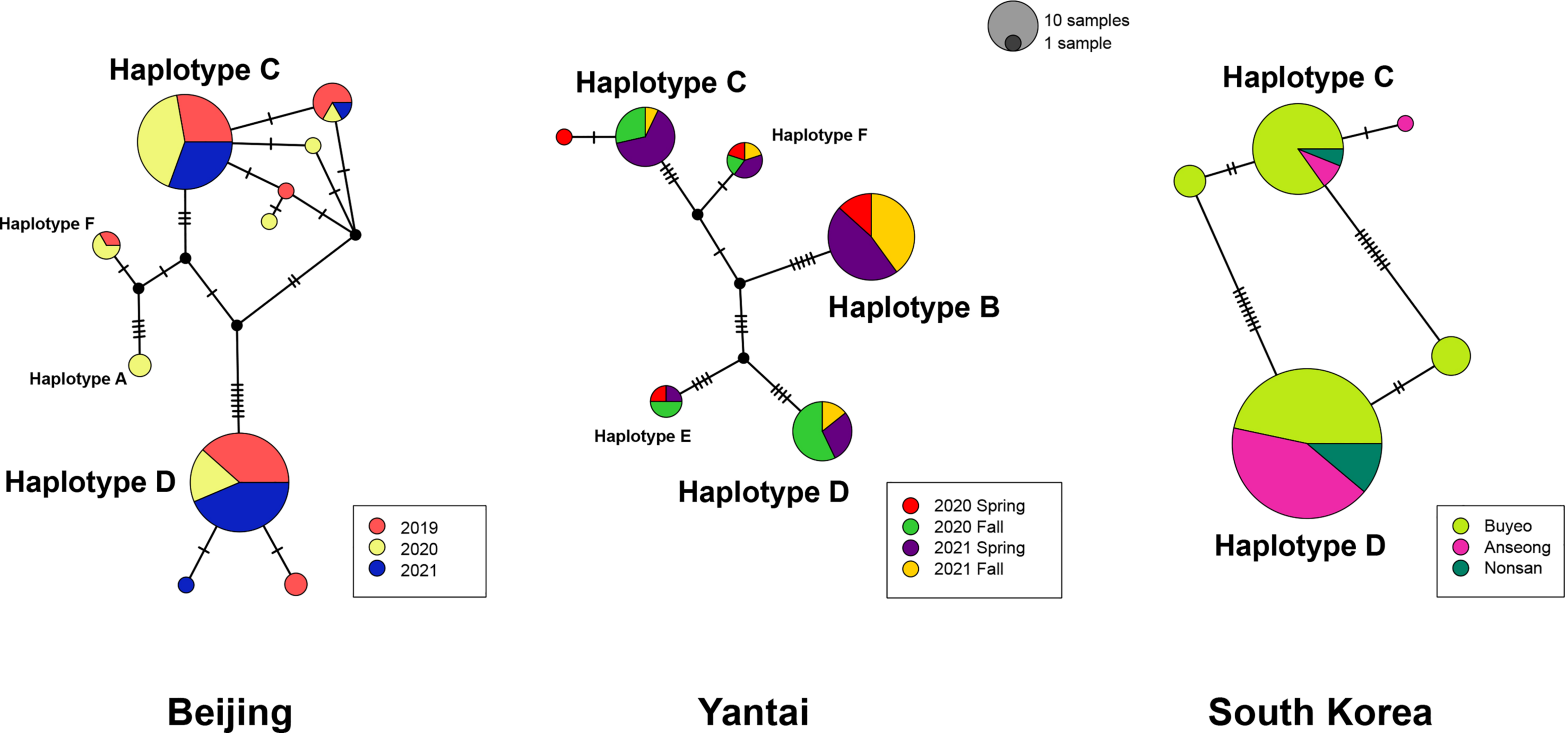
Statistical parsimony network of *A. orientalis* collected from Beijing, Yantai, and South Korea. Specimens are color-coded by collection year, emergence season, or location. Circle size is proportional to the number of samples, and all three networks are on the same scale. Each tick on branches represents a single mutational step.

In order to detect potential genetic differences between groups of specimens, which included multiple collecting years for Beijing, wasp emergence seasons for Yantai, and sampling locations for the South Korean population, those groups were labelled with separate colors in **Figure 2**. Among the Chinese samples, we observe no apparent association of haplotypes with sampling year or emergence season, although some year or emergence season categories recovered a greater number of haplotypes than others. Uncorrected *p*-distances within each sampling year or emergence season were close to distances between years or seasons (**Supplementary Table 2**). In Beijing, the majority of specimens belonged to Haplotype C or D and their derivatives, which differed from the respective main haplotypes by one or two substitutions. Collections made in different years had similar genetic composition. However, we did not find group B or E in Beijing, which seemed to be unique to the Yantai population. Indeed, Haplotype B was the most abundant haplotype among all Yantai specimens at a frequency of 44.12% (30/68), double the frequency of Haplotype C or D. However, Haplotype B was completely absent among all 15 wasps sampled from the fall 2020 emergence. In contrast to the high genetic diversity among Chinese specimens, the South Korean population had a much lower diversity and only possessed Haplotype C and D and a few derivatives, despite a sampling size close to China. Haplotype D is more prevalent than C in South Korea, occurring at a ratio of 1.5:1 in Buyeo, 5:1 in Nonsan, and up to 12.7:1 in Anseong. Together these two major haplotypes accounted for 91.8% (123/134) of sampled specimens. Interestingly, minor-frequency haplotypes found in the South Korean population were not recovered from the native populations in China.

### Rearing of iso-female lines

**Figure 3:**
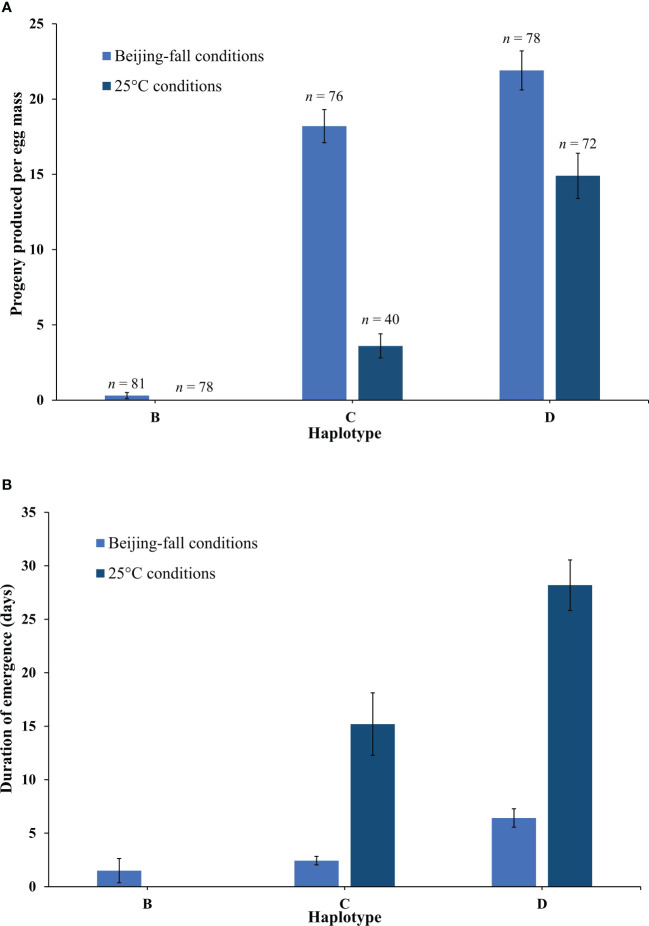
**(A)** The mean number of *Anastatus orientalis* progeny (female and males) produced per egg mass by Haplotypes (B, C, and D) in Beijing-fall conditions and 25°C long-day conditions, and **(B)** the number of days from the first wasp emergence date to the last wasp emergence date. The bars show means and standard errors. Because no Haplotype B wasps emerged in 25°C long-day conditions, no data on duration of emergence is available.

The emergence rate of *Anastatus orientalis* Haplotypes B, C, and D varied significantly (Full model F_5,419 = _99.59, p < 0.0001) in response to rearing in Beijing-fall and 25°C conditions (**Figure 3A**). Haplotype had the strongest effect (F = 185.31, p < 0.0001) followed by rearing condition (F = 76.08, p < 0.0001) and finally the interaction between these two factors (F = 23.04, p < 0.0001). Wasps presenting Haplotype B produced very few progeny in Beijing-fall conditions (0.3 ± 0.2 wasps per egg mass) and no progeny in 25°C conditions (i.e., they went into diapause). Wasps presenting Haplotype C produced a high number of progeny in Beijing-fall conditions (18.2 ± 1.1 wasps per egg mass) and a low number of progeny in 25°C conditions (3.6 ± 0.8 wasps per egg mass). Wasps presenting Haplotype D produced a high number of progeny in both Beijing-fall and 25°C conditions (21.9 ± 1.3 and 14.9 ± 1.5 wasps per egg mass, respectively). Overall, more female progeny emerged than male progeny, which is consistent with previous findings (10). There was no significant effect of haplotype, rearing condition, or their interaction on the resulting proportion of females (p = 0.16), though overall the proportion of females produced for Haplotype B was lower (49% female as compared to 81%–84% for Haplotype C and 73%–79% for Haplotype D). This is likely because neither rearing condition tested in this study was optimal for Haplotype B.

The timing of *A. orientalis* emergence also varied between the two rearing conditions (**Figure 3B**). Wasps began emerging later (first exposure date to first emergence date) in Beijing-fall conditions than in 25°C conditions (61.0 ± 0.5 and 43.0 ± 0.7 days to first wasp emergence, respectively). Wasps emerged over a shorter duration (first emergence date to last emergence date) in Beijing-fall conditions than in 25°C conditions and this differed by haplotype (F_4,236 = _51.03, p < 0.0001). Haplotype had the strongest effect (F = 23.26, p < 0.0001) followed by the interaction between haplotype and rearing condition (F = 6.54, p = 0.0112).

## Discussion

It has long been recognized that genetic variation has an impact on life history traits, even though the heritability of such traits may not be as strong as that of morphological traits (33). Intrigued by the contradictory observations of different diapause behaviors exhibited by *Anastatus orientalis* populations, we aimed to assess mitochondrial diversity within this parasitoid wasp—a potential biological control agent for the invasive spotted lanternfly, *Lycorma delicatula*—and the association between genetic variability and its life history traits. Our study contributes to the understanding of the biology of *A. orientalis*, which is critical for wasp rearing and subsequent tests of host specificity (34, 35).

Molecular analysis of mitochondrial data revealed considerable genetic variation by recovering six haplotype groups in *A. orientalis* collected from two locations in China (**Figure 1**). Two common Haplotypes C and D and a rare Haplotype F are shared between Beijing and Yantai, while the other three haplotypes (A, B, and E) seem to be restricted to either Beijing or Yantai (**Figure 2**). Sharing only a portion of haplotypes between those two locations that are 500 km apart, which likely exceed the dispersal limit of the wasp, could potentially be attributed to inadvertent transportation of the host egg mass by humans. It must be noted that the designation of haplotype groups is not based on a specific cutoff value of genetic distance, which varied between group pairs, but rather based on the relative separation of those groups on the ML tree. Interestingly, a comparison to the prior study that included fewer specimens but more sampling locations (20) revealed a generally consistent pattern, namely five out of the six haplotype groups identified here had corresponding representatives from that study, suggesting a comparable level of genetic divergence recovered between the two studies despite different sample sizes and origins. Together these findings indicate high levels of local genetic diversity which contrasts with the lack of a broad-scale phylogeographic structure in *A. orientalis*.

Maximum intraspecific divergence observed among the 160 Chinese specimens reached 1.56%, and the largest divergence between haplotype groups after controlling for within-group variation was 1.44% between groups B and D, which can occur in sympatry in Yantai. This level of mitochondrial divergence exceeds the 1% threshold for a confident species identification set by BOLD (36) but is similar to that of other widespread parasitoid wasps such as *Aphidius ervi* (37) and *Diaeretiella rapae* (38). We have examined morphological characters of both male and female wasps from the three major Haplotypes B, C, and D using taxonomic keys offered in the original description of *A. orientalis* (6) and a review of the genus in China (39). We did not find noticeable morphological differences among those groups, indicating that the diversity is cryptic in this species.

Although Beijing and Yantai populations share some haplotype groups, the main distinction between their genetic composition is the occurrence of Haplotype B unique to Yantai, which accounts for nearly half of sampled Yantai specimens. This haplotype seems to be specially adapted to Yantai conditions, whereas the other two common haplotype groups (C and D) are more tolerant to varied environmental factors. However, even between Haplotypes C and D we observed different responses in diapause behaviors to Beijing-fall conditions vs. the 25°C conditions. Due to space and time constraints, we were unable to evaluate haplotype responses under Yantai-associated conditions, which would provide an avenue for future study. Reports in the literature indicate that *A. orientalis* can be continuously reared at 25°C long-day conditions for multiple generations in China (6) and South Korea (9), but a recent study using specimens from Beijing found contradictory results (10). Based on results from our genetic analysis and iso-female line rearing, it is highly likely that specimens from the two earlier studies were mostly composed of Haplotype D, which is predominant in South Korea, and the colony used by Broadley et al. (10) were mainly Haplotype C. Indeed, we subsequently genotyped this colony and determined it was a homogenous Haplotype C colony (30).

*Anastatus orientalis* has been introduced into South Korea to control the invasive population of *L. delicatula*. Reduced genetic diversity of the Korean *A. orientalis* compared to native Chinese populations is in line with its introductory nature. However, the actual introduction history is somewhat complicated. The formal introduction was initiated in 2011 *via* an international cooperative project between the National Institute of Agricultural Sciences of Rural Development Administration (RDA) of South Korea and the Chinese Academy of Forestry in Beijing (8). Those wasps were therefore presumably originated from Beijing. But prior to the release of introduced wasps, *A. orientalis* had already been reported in South Korea from overwintering egg masses of spotted lanternfly collected in April 2010 (40). At that time the parasitoid wasp could not be accurately identified and was referred to as *Anastatus* sp. similar to *A. japonicus* (40), because only in 2015 was *A. orientalis* described as a new species (6). Considering these details, *A. orientalis* have been introduced 1) inadvertently with the invasive *L. delicatula*, which were first documented as a pest in South Korea in 2005 (41), and 2) purposely in large quantity through the international biological control effort in 2011. Currently, *A. orientalis* appears to be distributed throughout South Korea. This scenario is supported by the genetic data, as over 90% of Korean *A. orientalis* possessed either Haplotype C or D, which are also the most abundant haplotypes in Beijing. Additionally, the presence of some minor rare haplotypes not recovered from Beijing may suggest additional sources of introduction.

From this study, we have gained a better understanding of the genetic differentiation within and across populations of *A. orientalis* and how these relate to rearing specifications. We designed new species-specific COI primers and detected six distinct haplotype groups, some of which were regionally specific and some of which coexisted in the same geographic area. The expanded sampling and sequence data provides new insights into the genetic diversity of *A. orientalis* both in its native and introduced range. Additionally, by developing iso-female lines of the three most common of these haplotypes, we determined that the lines responded to the same conditions differently demonstrating the direct impact of genetics on diapause behaviors. These findings help to explain the contradictions in rearing methods presented in prior studies. This work is essential for differentiating what haplotype groups were evaluated in prior studies and for optimizing the laboratory rearing, evaluating annual life cycle characteristics, and testing host specificity of each *A. orientalis* haplotype separately.

## Data availability statement

The datasets presented in this study can be found in online repositories. The name of the repository and accession numbers can be found below: NCBI; OQ555811 - OQ556104.

## Author contributions

YW, HB, and JG designed the study. XW and LC provided the parasitoids for the study. YW, HB, KV, JM, HN, YK, CL, and AM ran the studies. YW, HB, JM, HN, YK analyzed the data. YW, HB, JM, and YK wrote the manuscript. All coauthors contributed feedback along the way and suggestions to manuscript drafts. All authors contributed to the article and approved the submitted version.
