# Supplementary material for: Cryptic genetic diversity and associated ecological differences of Anastatus orientalis, an egg parasitoid of the spotted lanternfly
Source: Front Insect Sci. 2023 Jun 2;3:1154651. doi: 10.3389/finsc.2023.1154651 (PMC10926478; doi:10.3389/finsc.2023.1154651)
Supplement: Supplementary file 1 [file Image_1.pdf]

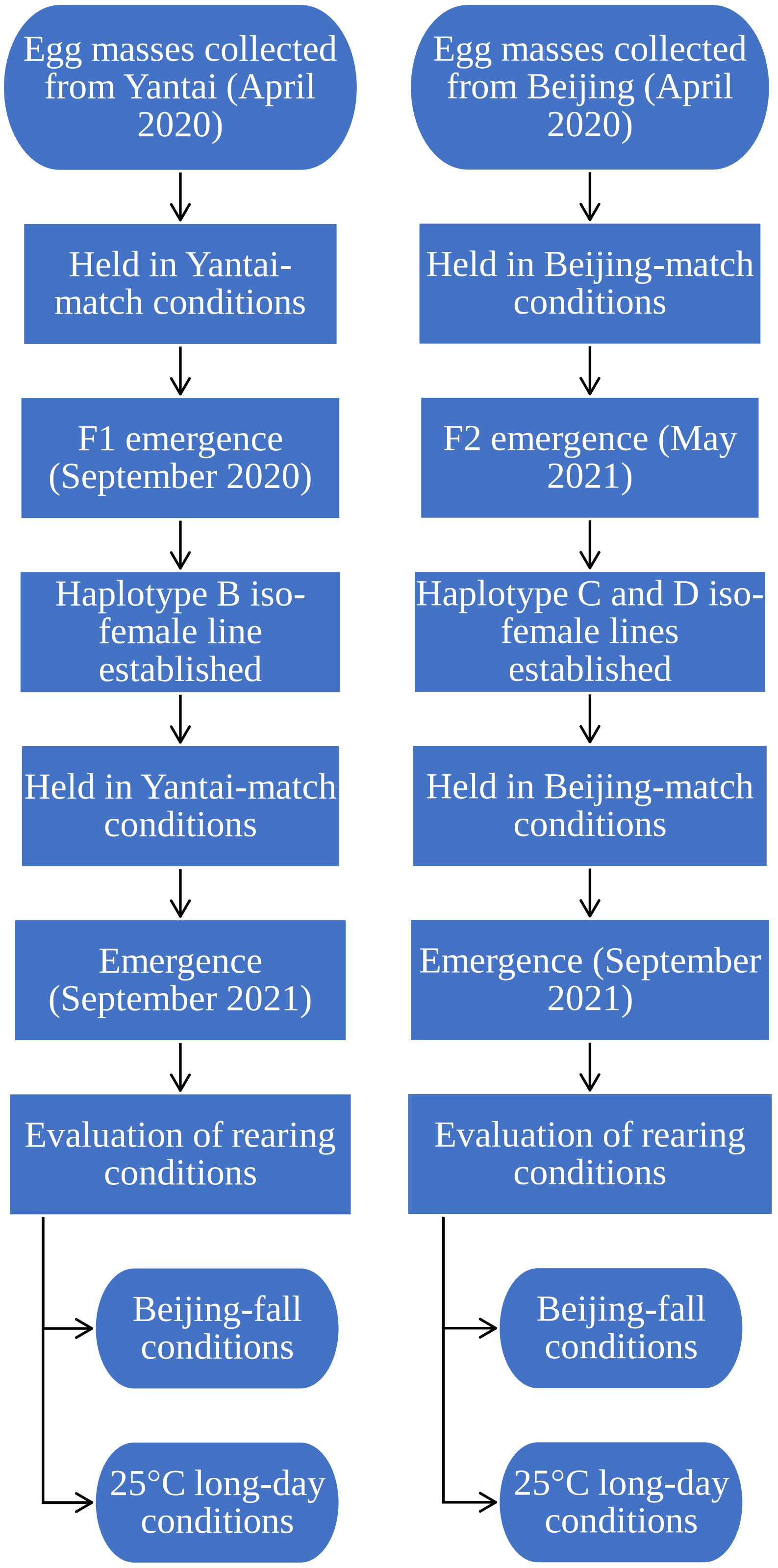

Supplementary Figure 1. A summary of the insect material collection, establishment of iso-female lines, and evaluation of rearing conditions conducted in this study.
